# Autosomal dominant optic neuropathy and sensorineual hearing loss associated with a novel mutation of WFS1

**Published:** 2010-01-12

**Authors:** Barend F.T. Hogewind, Ronald J.E. Pennings, Frans A. Hol, Henricus P.M. Kunst, Elisabeth H. Hoefsloot, Johannes R.M. Cruysberg, Cor W.R.J. Cremers

**Affiliations:** 1 Department of Ophthalmology, Radboud University Nijmegen Medical Centre, Nijmegen, The Netherlands; 2 Department of Otorhinolaryngology, Radboud University Nijmegen Medical Centre, Nijmegen, The Netherlands; 3 Department of Human Genetics, Radboud University Nijmegen Medical Centre, Nijmegen, The Netherlands

## Abstract

**Purpose:**

To describe the phenotype of a novel Wolframin (*WFS1*) mutation in a family with autosomal dominant optic neuropathy and deafness. The study is designed as a retrospective observational case series.

**Methods:**

Seven members of a Dutch family underwent ophthalmological, otological, and genetical examinations in one institution. Fasting serum glucose was assessed in the affected family members.

**Results:**

All affected individuals showed loss of neuroretinal rim of the optic nerve at fundoscopy with enlarged blind spots at perimetry. They showed a red-green color vision defect at color vision tests and deviations at visually evoked response tests. The audiograms of the affected individuals showed hearing loss and were relatively flat. The unaffected individuals showed no visual deviations or hearing impairment. The affected family members had no glucose intolerance. Leber hereditary optic neuropathy (LHON) mitochondrial mutations and mutations in the Optic atrophy-1 gene (*OPA1*) were excluded. In the affected individuals, a novel missense mutation c.2508G>C (p.Lys836Asn) in exon 8 of *WFS1* was identified.

**Conclusions:**

This study describes the phenotype of a family with autosomal dominant optic neuropathy and hearing impairment associated with a novel missense mutation in *WFS1*.

## Introduction

Hereditary optic neuropathy (HON) is a disease entity characterized by symmetric, bilateral, central visual loss with deviations of the papillomacular nerve fiber bundle resulting in cupping of the disk and in central or cecocentral scotomas and generalized constriction of the visual fields. In later stages, visual loss becomes severe, usually worse than 20/200 [1]. HON is seen in isolated autosomal dominant optic neuropathy, in Leber hereditary optic neuropathy (LHON; OMIM 535000), in Wolfram Syndrome (OMIM 222300), and in diseases with primarily neurologic or systemic manifestations such as hereditary ataxias, hereditary polyneuropathies, hereditary spastic paraplegias, hereditary muscular dystrophies, and storage diseases [2].

The combination of autosomal dominant optic neuropathy and deafness has been reported in families and in isolated cases with a heterozygous missense mutation in Optic atrophy-1 gene *(OPA1*; OMIM 605290) [3–6]. Eiberg et al. [7] described a Danish family who had autosomal dominant optic neuropathy and deafness caused by a mutation in the Wolframin *(WFS1)* gene. *WFS1*, on chromosome 4p16.3, contains eight exons. Mutations in this gene are reported to be responsible for Wolfram Syndrome, Deafness Autosomal dominant type 6/14 (DFNA6/14; OMIM 600965, a low-frequency sensorineural hearing loss that is inherited in an autosomal dominant manner), psychiatric disorders, and diabetes mellitus (OMIM 606201). After examination of the family with autosomal dominant optic neuropathy and deafness, Eiberg et al. [7] concluded that the patients also had impaired glucose tolerance. Valéro described a French family with the same missense mutation [8]. There were only two affected individuals: the proband and his mother suffered diabetes mellitus with congenital hearing loss. At the age of 60 the mother was diagnosed with optic atrophy. The present report describes the phenotype of a third family with autosomal dominant optic neuropathy and deafness that is associated with a novel missense mutation in *WFS1*.

## Methods

**Figure 1:**
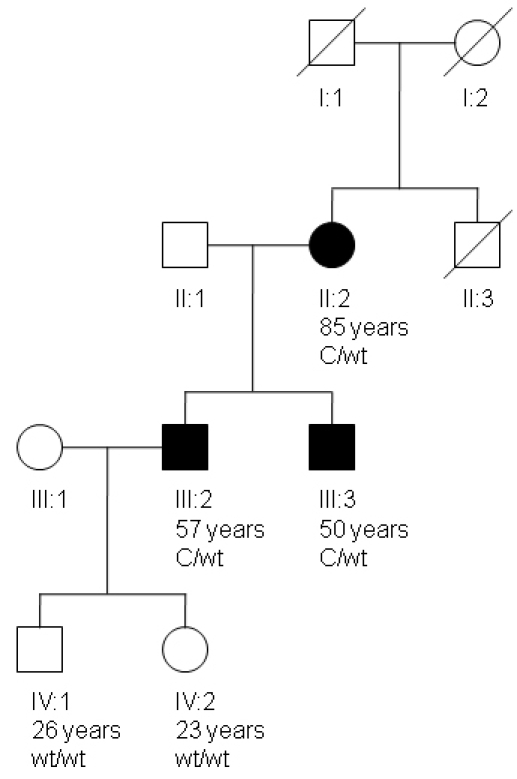
The p.Lys836Asn mutation in the studied family with autosomal dominant optic neuropathy and hearing impairment. In the pedigree the individual number, the age of the individual, and the *WFS1* genotypes are depicted. The affected woman and her two sons suffer optic neuropathy and hearing impairment and carry the c.2508G>C *WFS1* mutation. Abbreviations: c.2508G>C *WFS1* mutation (C); wildtype allele (wt).

The proband, a 57-year-old man, was referred to our tertiary referral hospital for progressive hearing loss, which coexisted with optic neuropathy. The question was raised whether he was a good candidate for cochlear implantation. Medical history showed that both his mother and brother also had optic neuropathy and hearing loss. Figure 1 shows the pedigree of this family. Informed consent was obtained from both the proband and his family to participate in this study. The research adhered to the tenets of the Declaration of Helsinki and was approved by the Institutional Review Board (Commissie Mensgebonden Onderzoek), Radboud University Nijmegen Medical Centre, Nijmegen, The Netherlands.

All family members were examined in our outpatient clinic and medical history was taken. For all family members except patient II:2, the ophthalmological examinations included best corrected visual acuity measurements, slit-lamp microscopy and ophthalmoscopy. Goldmann perimetry was performed to evaluate visual field size. A morphometric analysis of the optic disc was performed using the Heidelberg Retina Tomograph II (HRT; Heidelberg Engineering, Heidelberg, Germany) [9]. Color vision was assessed with the Hardy-Rand-Ritter (HRR) pseudoisochromatic plates, the Lanthony new color test, the Neitz anomaloscope (Neitz, Tokyo, Japan), and the standard pseudoisochromatic plates test. In addition, visually evoked potentials (VEP; Roland Consult, Brandenburg, Germany) were evaluated. Patient II:2 only underwent standard ocular examinations. All individuals underwent pure-tone audiometry and speech audiometry. Otoscopy was performed on all family members to rule out middle ear pathology. As part of a preoperative selection procedure for cochlear implantation, the proband also underwent electronystagmography, computed tomography (CT), and auditory steady-state response (ASSR) testing.

The affected family members underwent fasting serum testing to exclude diabetes mellitus (serum specific insulin, serum C-peptide and HbA_1_C were analyzed in the proband and plasma glucose was analyzed in all affected individuals).

Blood samples of all living individuals were collected in EDTA tubes and kept at room temperature. DNA was isolated within five days after withdrawal on a Chemagen MSM1 platform using the Chemagic DNA blood 10k kit (Chemagen, Baesweiler, Germany). Mutation analysis of *WFS1* was performed by direct sequencing of the entire coding region (exon 2 to 8). The coding exons and the flanking intronic sequences were PCR amplified and subsequently sequenced on a 3730 automated sequencer using Dye terminator chemistry (Applied Biosystems, Foster City, CA). For primer information and PCR conditions see Table 1. In addition, *OPA1* and three known LHON mutations (mtDNA positions m.11778, m.3460, and m.14484) were screened by a combination of dHPLC (Transgenomic, Inc., Omaha, NE) and direct sequencing analysis.

**Table 1:** *WFS1* primers.

**Exon**	**Forward primer (5′-3′)**	**Reverse primer (5′-3′)**
2	TGTCTCCAGCAGACACTAAG	GGGTGGCTGAACCCCGTTC
3	GAAGACCCTCATGCCTTGTC	ATCTCAGGCACCGACACTTC
4	GGAGAATCTGGAGGCTGACT	ACAAGCTGCTCAACCCTCCA
5	AGAGTGGCACCGAAACCA	TCCTGTGGGAAGACCCAG
6	AACAGTGCGCCAGTTTCTG	GAGGCACGGGTGAGATAGG
7	CCCATTGCTCTGTGTGAGG	GAAGGTGCCCTGCCTGAG
8–1	AGAGGCAGGGTGGTCAGAG	GAGAGCAGGAAATGGGCATA
8–2	AGAACTTCCGCACCCTCAC	AGGTAGGGCACAAGGTAGCA
8–3	TATCTCTTCTTCCGCATGGC	TACTGCTGCCAGGTCAGTGT
8–4	CAAGCTCATCCTGGTGTGG	GTGACGTCGTCCTCCTCG
8–5	CCCTGCCACATCAAGAAGTT	GGTCTCTGCAGCCACAGTCT

The table shows the forward and reverse primers used for PCR amplification of the coding exons of the WFS1 gene. Exons 2 to 7 were amplified as single fragments, whereas the larger exon 8 was amplified in 5 overlapping fragments. PCR was performed using AmpliTaq Gold 360 Master mix (Applied Biosystems, Foster City, CA) with 50 ng genomic DNA and 10 pmol of each primer in a total volume of 25 µl. PCR reaction conditions for all fragments were: 95 °C for 10 min; then 35 cycles of 95 °C for 30s, 60 °C for 1 min and 72 °C for 30s, followed by 7 min at 72 °C.

## Results

### Medical history

From the pedigree in Figure 1, it can be concluded that the disease has an autosomal dominant or mitochondrial inheritance pattern. Neither the maternal grandparents nor the uncle of the proband had hearing impairment. All affected individuals had no symptoms additional to progressive sensorineural hearing impairment and optic atrophy. No other Wolfram syndrome-related symptoms (diabetes mellitus, diabetes insipidus, renal or psychiatric problems) were mentioned during each participant’s history and physical examination.

### Ophthalmological results

**Figure 2:**
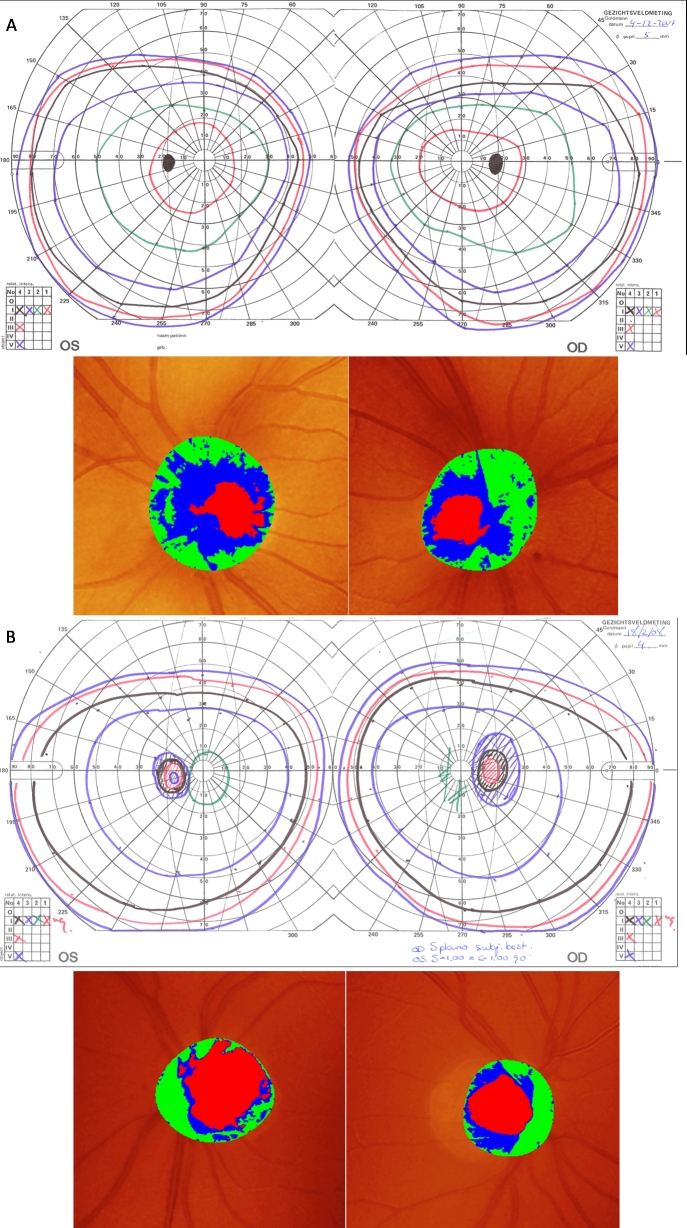
Phenotypic characterization of two members of a Dutch family. **A**: The upper portion presents the visual field printouts of the Goldmann perimetry (GP) test, and the lower part shows screenshots from Heidelberg Retina Tomograph II (HRT). The right and left column correspond to the right and left eye of individual IV:2, respectively. The small temporal black area in the GP corresponds to the physiologic blind spot. HRT scans demonstrate a physiologic optic disc cupping measure (red areas) and a physiologic thinned neuroretinal rim (blue-green area) of the optic disc. **B**: GP and HRT results for III:3 are shown. The large dark areas in the GP correspond to the enlarged blind spot due to optic neuropathy. HRT scans demonstrate increased optic disc cupping measure (red areas) and a thinned neuroretinal rim (blue-green area) of the optic disc.

The results of the ophthalmological examinations are shown in Table 2 and Figure 2. It should be noted that not all the family members were examined ophthalmologically and that the unaffected individuals that were examined ophthalmologically were of a younger generation than the affected individuals. All affected individuals had an enlarged blind spot at Goldmann perimetry, loss of neuroretinal rim on HRT, and a deviating VEP. They also showed an indication of protan and deutan axes (i.e., a red-green defect) at color vision testing. The unaffected individuals did not have an enlarged blind spot at Goldmann perimetry, loss of neuroretinal rim on HRT, nor a deviating VEP.

**Table 2:** Ophthalmological examination results

**Patient**	**Age (Y)**	**Sex**	**Mutation reported in *WFS1***	**AoO (Y)**	**Eye**	**BCVA**	**Media**	**Papilla**	**Visual field**	**VEP**	**Color vision**	**HRT**
II:2	85	F	+	30	RE	0.1	Clear	Optic disc atrophy	Na	Na	Na	Na
					LE	0.05	Clear	Optic disc atrophy	Na	Na	Na	Na
III:2	57	M	+	11	RE	0.1	Clear	Optic disc atrophy	Enlarged blind spot	Delayed response	Marked protan and deutan axes	Outside normal limits
					LE	0.2	Clear	Optic disc atrophy	Enlarged blind spot	Delayed response	Marked protan and deutan axes	Outside normal limits
III:3	50	M	+	10	RE	0.2	Clear	Optic disc atrophy	Enlarged blind spot	Delayed response	Marked protan and deutan axes	Outside normal limits
					LE	0.4	Clear	Optic disc Atrophy	Enlarged blind spot	Delayed response	Marked protan and deutan axes	Outside normal limits
IV:1	26	F	-	11	RE	1.6	Clear	Normal	No significant alterations	Intact	Normal	Normal
					LE	1.2	Clear	Normal	No significant alterations	Intact	Normal	Normal
IV:2	23	M	-	11	RE	1.6	Clear	Normal	No significant alterations	Intact	Normal	Normal
					LE	1.6	Clear	Normal	No significant alterations	Intact	Normal	Normal

For the patients the age in years (Y) is shown as well as whether they carry the WFS1 mutation and their age of onset of ophthalmological complaints (AoO). The results of ophthalmological examination tests are depicted for each eye separately. The mutation carrying individuals all have optic disc atrophy and decreased results of Snellen test for best corrected visual acuity (BCVA). The results of the tests for visual field, visually evoked potentials (VEP), color vision and Heidelberg Retinal Tomography (HRT) were impaired for the affected individuals. The not affected individuals showed normal results for these tests. Abbreviations: female (F); male (M); right eye (RE); left eye (LE); not available (Na).

### Hearing results

**Figure 3:**
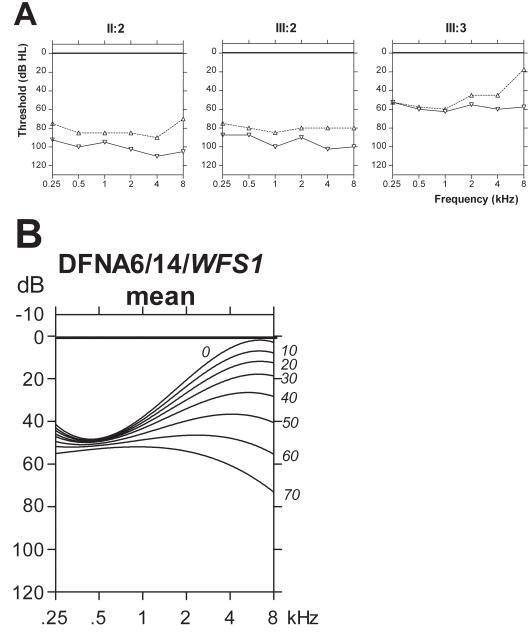
Audiograms of individuals with the p.Lys836Asn mutation in the *WFS1* gene. The mean pure-tone audiograms of right and left ear in affected persons II:2 (age 60 and 85 years), III:2 (age 52 and 57 years) and III:3 (age 37 and 51 years) are depicted in panel **A**. Dotted lines and upward triangular symbols indicate first available and straight lines, and downward triangular symbols indicate last-visit mean audiogram. The mean age-related typical audiograms for DFNA6/14, adapted and modified from Pennings and coworkers [29], are depicted in panel **B**.

The results of otological and audiological examinations are shown in Table 3. Otoscopy showed no abnormalities in all patients. Hearing was normal in both children (IV:1 and IV:2) of the proband and also normal (for his age) in the only living uncle (II:3) of the proband. For all affected individuals, the first available and last-visit pure-tone audiograms are shown in Figure 3, upper panel. They demonstrate a relatively flat-type of hearing loss at last visit. Remarkably, hearing in both the proband and his mother is so diminished that they hardly have any speech recognition. Individual III:3, the youngest of these three affected participants, still has relatively normal maximum speech recognition scores. His first available pure-tone audiogram demonstrated a typical low-frequency sensorineural hearing loss. No progression of hearing loss could be deduced from the pure-tone audiograms of study participants II:2 and III:2.

**Table 3:** Otological and audiological examination results

**Patient**	**Age (Y)**	**Sex**	**Mutation reported in WFS1**	**AoO (Y)**	**Otoscopy**	**FI RE/LE (dB HL)**	**MSRS RE/LE (%)**	**ENG**	**ASSR**	**CT temporal bone**
II:2	85	F	+	9	Normal	98/100	3/0	Na	Na	Na
II:3	75	M	-		Normal	10/12	Na	Na	Na	Na
III:1	57	F	-		Normal	22/18	100/100	Na	Na	Na
III:2	57	M	+	8	Normal	90/97	15/0	Normal	Conform pure tone audiogram	No anatomic abnormalities
III:3	51	M	+	14	Normal	58/60	93/90	Na	Na	Na
IV:1	26	F	-		Normal	5/7	Na	Na	Na	Na
IV:2	23	M	-		Normal	18/20	100/100	Na	Na	Na

For the patients the age in years (Y) is shown as well as whether they carry the WFS1 mutation and their age of onset of audiological complaints (AoO). The results of the audiological examination tests are depicted for each ear separately. The ears of the mutation carrying individuals have an increased Fletcher Index (FI; mean threshold for 0.5, 1, and 2 kHz) and a decreased maximum speech recognition score (MSRS). Abbreviations: female (F); male (M); right ear (RE); left ear (LE); decibel hearing loss (dB HL); electronystagmography (ENG); auditory steady-state response (ASSR); computed tomography (CT); not available (Na).

The proband had normal vestibular function. CT scan of the temporal bone showed no anatomic abnormalities. Central causes of sensorineural hearing loss were excluded by ASSR testing, which revealed normal function of the auditory nerve. The proband underwent successful right ear cochlear implantation with a 22-electrode implant (Cochlear; Nucleus Freedom, Sidney, Australia). Seven months after implantation, he was found to have 83% speech recognition at 70 dB sound pressure level (SPL). Prior to implantation, aided thresholds with a conventional aid on the right were tested: no speech recognition was found at 70 dB SPL and the maximum speech recognition of about 20% was found at 80 dB SPL.

### Genetic results

**Figure 4:**
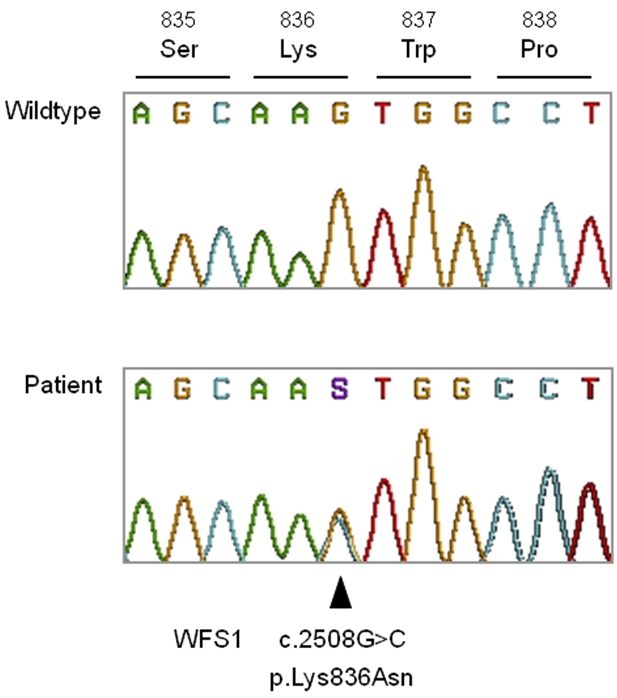
The p.Lys836Asn mutation in the *WFS1* gene. The figure shows results of the DNA sequence analysis of part of exon 8 of the *WFS1* gene. The upper chromatogram exhibits the wildtype sequence whereas the lower chromatogram shows the sequence of an affected family member. The arrowhead marks the nucleotide at position 2508. The results show that the patient heterozygous for a guanine (G; yellow peak) to cytosine (C; blue peak) exchange at this position, which translates into a lysine to asparagine amino acid substitution at position 836 of the corresponding protein.

**Figure 5:**
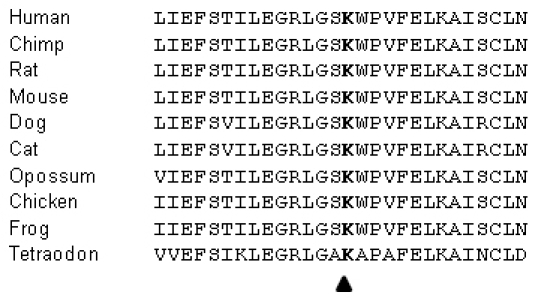
Evolutionarily conservation of the lysine residue at position 836 of the WFS1 protein. Evolutionarily conservation of the lysine residue at position 836 of the WFS1 protein was assessed through multiple alignment of WFS1 protein sequences derived from different species. The arrowhead marks the lysine residue (K) at position 836. The multiple sequence alignment shows that the lysine is highly conserved in the different species which provides evidence that this residue is important for proper protein functioning.

Screening of mtDNA positions m.11778, m.3460 and m.14484 demonstrated that the three most frequent LHON mutations were not present. Screening of *WFS1* and *OPA1* revealed no mutations in all coding regions of *OPA1*. In exon 8 of *WFS1,* a heterozygous mutation was identified in all three affected patients. This variant was not detected in the unaffected family members. At position 2508, a substitution of a cytosine for a guanine (c.2508G>C) leads to the amino acid substitution p.Lys836Asn. Based on the medical history and clinical information available, we assume that this is a de novo mutation. Figure 4 shows the normal and mutated sequences. We believe this variant to be pathogenic because the mutated lysine is evolutionarily highly conserved (Figure 5), is located in a conserved region of the protein, and cosegregates with the disease in this family. In addition, this variant has never been identified in our laboratory (so far we analyzed the WFS1 sequence in 200 European patient chromosomes).

### Exclusion of diabetes mellitus

Diabetes mellitus was excluded in the affected individuals by assessing fasting serum glucose (all within the reference value 4.0–5.6 mmol/l) and HbA_1_C (all within the reference value 4.2%–6.3%). In the proband, insulin (9 mE/l, reference value 8–20) and C-peptide (0.62 mmol/l, reference value 0.17–1) were assessed as well, and found to be in the normal range.

## Discussion

In this article we report a novel missense mutation (p.Lys836Asn) in exon 8 of *WFS1* that is associated with autosomal dominant optic neuropathy and deafness. The disease shows 100% segregation with the mutation. The three affected individuals did not have diabetes mellitus or any other symptoms. Since there was no autosomal recessive inheritance, they did not present with Wolfram syndrome spectrum. They also did not have DFNA6/14, the nonsyndromic autosomal dominant low frequency sensorineural hearing loss that is caused by *WFS1* mutations. The affected participants did show more severe sensorineural hearing loss with involvement of all frequencies, and they did have optic atrophy.

### Hereditary optic neuropathy

HON is a clinically and genetically heterogeneous condition. Isolated autosomal dominant optic neuropathy is caused by four genes: *OPA1, OPA3, OPA4,* and *OPA5*. *OPA1* (OMIM 165500) is the most frequently encountered type of isolated autosomal dominant optic neuropathy. Symptoms usually manifest in the first decade of life, and, in most cases, visual impairment develops gradually over many years. The reduction in visual acuity tends to be mild or moderate [10,11]. Visual field defects mainly involve the central portion of the field and include cecocentral scotomas, paracentral defects, and pseudobitemporal defects. Neuroretinal rim pallor in autosomal dominant optic neuropathy is most pronounced temporally, but usually involves the entire optic disc [10].

Optic neuropathy is also a sequela of LHON. LHON is the result of maternal mitochondrial DNA mutations. Visual loss usually begins painlessly and centrally in one eye, and the second eye is affected weeks to months later with an acute or subacute course. However, the duration of progression of visual loss in each eye varies and may be difficult to document accurately. Eventually, optic atrophy bundle supervenes [2].

Optic neuropathy is also one of the main symptoms in Wolfram syndrome. Caused by mutations in *WFS1* on chromosome 4p16.3, Wolfram syndrome is an autosomal recessive neurodegenerative syndrome [12]. The minimal criteria for diagnosis are diabetes mellitus and optic atrophy [1]. Sensorineural hearing loss is often an additional finding.

### Autosomal dominant optic neuropathy and deafness

The affected individuals of the described family have severe optic nerve damage as well as sensorineural hearing loss that is inherited in an autosomal dominant way. In 1977, Deutman presented a family with autosomal dominant optic neuropathy and deafness similar to our family: the affected individuals had sensorineural hearing impairment and optic atrophy but no other symptoms. All affected individuals had hearing loss with a relatively flat audiogram, an enlarged blind spot at Goldmann perimetry, deutanomaly, and a deviating VER [13]. There was no information available on the genetic background of this family.

Autosomal dominant optic neuropathy and deafness has also been reported in several families and in isolated cases with a heterozygous missense mutation (p.Arg445His or p.Gly439Val) in *OPA1* [3–5,14]. In our family, the presentation of the optic atrophy and the bilateral progressive sensorineural hearing loss is similar to the presentation of the families with autosomal dominant optic neuropathy and deafness and a mutation in *OPA1*. However, *OPA1* mutations were excluded by sequencing analysis. In addition, the *OPA1* mutations seem to cause a broader phenotype with ptosis, ophthalmoplegia, ataxia, axonal sensory-motor polyneuropathy, and mitochondrial myopathy [5,14,15]. Another *OPA1* mutation (p.Tyr582Cys) is responsible for progressive hearing loss that necessitated cochlear implantation, macrocytic anemia, and hypogonadism [16]. Interestingly, the patient of this case had progressive external ophthalmoplegia and central vision loss. Because no optic pallor was seen on fundoscopy, it was believed the patient’s vision loss was not caused by optic atrophy.

The combination of hearing loss and LHON has not been ascribed to a single mutation so far [17]. However, Hofmann and coworkers [18] proposed that LHON mutations represent a susceptibility factor for Wolfram syndrome which, by interaction with further exogeneous or genetic factors, might increase the risk for disease. In our family, the main LHON mutations were excluded by sequencing analysis. The phenotype of LHON mutations is characterized by an acute or subacute loss of visual acuity with changes of the optic disc. None of the affected family members suffered such a period.

### Wolframin

*WFS1* encodes for wolframin, a protein known to contain nine predicted transmembrane domains. So far, about 110 mutations in *WFS1* are believed to cause the Wolfram syndrome [19–23]. Eiberg [7] and Valéro [8] reported two families who had a *WFS1* missense mutation (p.Gln864Lys) in the same conserved region as the mutation that we found. This mutation caused an autosomal dominant clinical triad: congenital hearing impairment, diabetes mellitus, and optic atrophy. In the study by Valéro, however, the proband (thus far) had no optic atrophy [8]. Mutations in *WFS1* are also responsible for other conditions such as psychiatric disorders and diabetes mellitus (Lesperance laboratory database). The pleiotropy of this disorder can possibly be explained by alternative splicing: missense mutations would occur in regions that are spliced out in specific organs. Challenging *WFS1* expression studies of these organs would be needed to prove or reject this hypothesis.

To date, 26 *WFS1* mutations have been reported to cause DFNA6/14 [21–24], an unusual type of hearing loss that affects frequencies at 2,000 Hz and below [25–27]. In general, hearing loss in DFNA6/14 is not progressive, however, some families were reported to have progressive hearing loss that could be attributed to presbycusis [28,29]. Because high frequency hearing is generally preserved, DFNA6/14 patients retain excellent understanding of speech, although presbycusis may cause high-frequency hearing loss later in life. Consequently, DFNA6/14 in younger patients is often asymptomatic, and many patients choose not to wear hearing aids. This contrasts with the affected family members in the current family who showed a flat-type of hearing loss with poor speech recognition.

Due to a lack of previous audiograms, it was difficult to evaluate progression of hearing loss in II:2 and III:2. It should however be noted that the first available audiograms of II:2 were made at the age of 60 years and that the first available audiograms of III:2 were made at the age of 60 years. Progression of their hearing loss may have occurred before these ages. Interestingly, the first pure-tone audiogram of III:3, done at the age of 38 years, shows a more pronounced loss at the lower frequencies, resembling the hearing loss characteristic of DFNA6/14 (see Figure 3, upper and lower panel).

Individual III:3 typically had low-frequency sensorineural hearing loss that resembled DFNA6/14 at the age of 38 years. In the following years, he experienced progression of his hearing loss at 8 kHz (40 dB) that appears to be bigger than the mean deterioration (approximately 1 dB/year) that was reported for this frequency based on analyses in several families with DFNA6/14 [28]. The hearing impairment in his brother, III:2, and his mother, II:2, is too profound for DFNA6/14. Thus it can be concluded that the phenotype of this novel *WFS1* mutation is more severe when compared to DFNA6/14. Hearing appears to deteriorate more progressively for the *WFS1* mutation and these patients also develop optic neuropathy.

### Pathogenesis of optic neuropathy due to *WFS1* mutations

The distribution of wolframin in the mammalian visual system, and the pathogenesis of optic atrophy due to mutations in *WFS1* remain unclear. Expression studies, however, have assessed the presence of wolframin in retinal ganglion cells and optic nerve glia cells of the cynomolgus monkey [30]. In rodents, the presence of mRNA and wolframin have been examined in the retina (amacrine cells, Müller cells, photoreceptors, horizontal cells, bipolar cells and retinal ganglion cells), in the optic nerve (particularly in astrocytes), in the optic tract, and in the brain (the superior colliculus, the dorsomedial part of the suprachiasmatic nucleus and layer II of the primary and secondary visual cortices) [31]. Kawano and coworkers hypothesized that mutant wolframin may contribute to the dysfunction of wolframin-expressing neurons as well as glial cells, which, in turn, may lead to optic neuropathy [31].

### Pathogenesis of deafness due to *WFS1* mutations

The function of wolframin in the inner ear and the mechanisms by which missense mutations cause hearing loss have not been extensively explored [32]. The expression of wolframin has been localized to the mouse cochlea at different developmental stages and is widely distributed in different cochlear cell types, including inner and outer hair cells, a variety of supporting cells, and cells of the lateral wall, spiral ganglion, and vestibule [33]. A similarity has been observed between wolframin expression and the presence of the canalicular reticulum, a specialized form of endoplasmic reticulum that is believed to be involved in transcellular ion transport [34]. Thus, wolframin may be involved in regulation of inner ear ion homeostasis as maintained by the canalicular reticulum [33,34]. The majority of causative DFNA6/14 mutations have been identified in exon 8, which contains the conserved C-terminal domain. This domain seems to have a crucial function in the cochlea [22,33], and the p.Lys836Asn mutation is also located in this domain. Because the proband of our study family highly benefits from his cochlear implant, this suggests that indeed there is a deleterious effect of the present *WFS1* mutation in the cochlea without any neurodegenerative symptoms that are so common in Wolfram syndrome. It is still not clear why DFNA6/14 patients show a stable low frequency hearing impairment with minor progression and why the affected family members in our study have such severe progression and profound hearing impairment. Interestingly, in our study family with visual impairment by optic neuropathy, there were no signs that the hearing impairment is caused by auditory neuropathy. According to the literature [35], most patients with Wolfram syndrome do not have auditory neuropathy, but most have sensorineural hearing loss caused by degeneration of the organ of Corti. In addition, patients with missense mutations in *WFS1*, causing DFNA6/14, also have sensorineural hearing loss and no auditory neuropathy. Apparently, this specific missense mutation in *WFS1* has a different effect in the inner ear than in the eye. Further studies are needed to elucidate the underlying pathogenetic mechanism [36].

### Conclusion

The results of our study suggest that p.Lys836Asn is a novel mutation in *WFS1* that is associated with autosomal dominant optic neuropathy and finally a severe to profound hearing loss with relatively flat audiograms. The present phenotype is similar to the phenotype caused by the only other reported *WFS1* missense mutation (p.Gln864Lys) that causes autosomal dominant optic neuropathy and deafness. However, some of those patients also have impaired glucose intolerance [7], and one patient has no optic neuropathy [8]. It is also similar to the phenotype of *OPA1* mutations causing autosomal dominant optic neuropathy and deafness, however, in general these mutations cause a more extensive phenotype that includes ptosis, ophthalmoplegia, ataxia, axonal sensory-motor polyneuropathy, mitochondrial myopathy, macrocytic anemia, and hypogonadism [6,14–16]. The symptoms in our study family also clearly differ from the clinical presentation of LHON, Wolfram syndrome, and DFNA6/14. On the basis of this study we advise to perform extensive genetic testing of at least *WFS1* and *OPA1* in cases of autosomal dominant optic neuropathy and deafness.
